# Supplementary material for: The Repurposed Drugs Suramin and Quinacrine Cooperatively Inhibit SARS-CoV-2 3CLpro In Vitro
Source: Viruses. 2021 May 10;13(5):873. doi: 10.3390/v13050873 (PMC8170883; doi:10.3390/v13050873)
Supplement: Supplementary file 1 [file viruses-13-00873-s001.zip › viruses-1190244-supplementary.pdf]

Supplementary Information

The repurposed drugs Suramin and Quinacrine cooperatively inhibit SARS-CoV-2 3CL^{pro} *in vitro*

Raphael J. Eberle[1,2#*], Danilo S. Olivier[3], Marcos S. Amaral[4], Ian Gering[1], Dieter Willbold[1,2,5], Raghuvir K. Arni[6], Monika A. Coronado[1,6#*]

[1]Institute of Biological Information Processing (IBI-7: Structural Biochemistry), Forschungszentrum Jülich, Jülich, Germany.

[2]Institut für Physikalische Biologie, Heinrich-Heine-Universität Düsseldorf, Universitätsstraße, Düsseldorf, Germany.

[3]Federal University of Tocantins, Araguaína-TO, Brazil.

[4]Institute of Physics, Federal University of Mato Grosso do Sul, Campo Grande-MS, Brazil.

[5]JuStruct: Jülich Centre for Structural Biology, Forchungszentrum Jülich, Jülich, Germany.

[6]Multiuser Center for Biomolecular Innovation, IBILCE, Universidade Estadual Paulista (UNESP), São Jose do Rio Preto-SP, Brazil.

[#]both authors contributed equally

*Correspondence to eberleraphael@gmail.com or monikacoronado@gmail.com

Table of contents

Supplementary Figure S1. Purification of SARS-Cov-2 3CL^{pro} and quality check of the produced protein.
Supplementary Figure S2. K_M determination of SARS-CoV-2 3CL^{pro}.
Supplementary Figure S3. Effect of Triton X-100 on the Quinacrine and Suramin inhibition against 3CL^{pro}.
Supplementary Figure S4. H_2O_2 generating capacity of Quinacrine and Suramin under the influence of 1mM TCEP.
Supplementary Figure S5. Quinacrine and Suramin with inhibitory activity against SARS-CoV-2 3CL^{pro}.
Supplementary Figure S6. Fluorescence spectroscopy of Trp at 295 nm of SARS-CoV-2 3CL^{pro} in the presence of the Suramin and Quinacrine.
Supplementary Figure S7. K_D determination of Quinacrine binding to SARS-CoV-2 3CL^{pro} using SPR.
Supplementary Figure S8. K_D determination of Suramin binding to SARS-CoV-2 3CL^{pro} using SPR.
Supplementary Figure S9. K_D determination of Quinacrine binding to SARS-CoV-2 3CL^{pro} under the influence of Suramin using SPR.
Supplementary Figure S10. Clustering analysis of the SARS-CoV-2 3CL^{pro} structure after MD simulation.
Supplementary Figure S11. Time dependent modifications of the SARS-CoV-2 3CL^{pro} dimer.
Supplementary Figure S12. SARS-CoV-2 3CL^{pro} dimeric structure after MD simulation.
Supplementary Figure S13. Time dependent modifications of SARS-CoV-2 3CL^{pro} dimer in the presence of Quinacrine in each active site.
Supplementary Figure S14. Time dependent modifications of SARS-CoV-2 3CLpro dimer in the presence of Suramin. Supplementary Figure S15. Atom numbers of Quinacrine and Suramin.
Supplementary Figure S16. Quinacrine and Suramin combination with inhibitory activity against SARS-CoV-2 3CL^{pro}.
Supplementary Figure S17. Time dependent modifications of SARS-CoV-2 3CL^{pro} dimer in the presence of Quinacrine and Suramin.
Table S1. K_D values of Quinacrine and Suramin SARS-CoV-2 3CL^{pro}, determined by SPR.
Table S2. Contributing amino acids in the substrate-binding sites of SARS-CoV-2 3CL^{pro}.
Table S3. SARS-CoV-2 3CL^{pro} Residues involved in forming H-bonds and hydrophobic contacts with Quinacrine, Suramin and Quinacrine + Suramin.
Table S4. Affected proteins by mutations in SARS-CoV-2 variants.

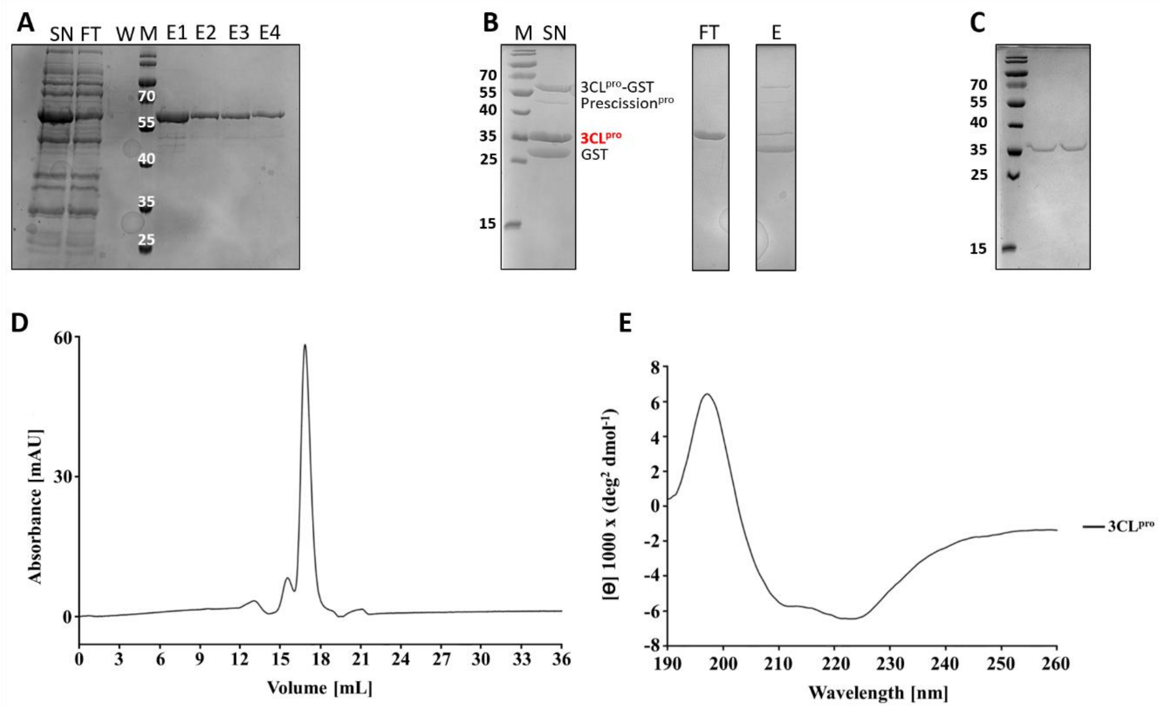

Figure S1. Purification of SARS-Cov-2 $3CL^{pro}$ and quality check of the produced protein. (A) SDS 15% Gel of $3CL^{pro}$_GST purification with GSH sepharose. SN: supernatant, FT: flow through, W: washing step, M: protein marker, E1-E4: Elution. **(B)** SDS 15% Gel of the $3CL^{pro}$_GST fusion protein cleavage by $PreScission^{pro}$. M: protein marker. SN: cleaving approach, containing $3CL^{pro}$_GST, $PreScission^{pro}$, $3CL^{pro}$ and GST. FT: contain $3CL^{pro}$, E: elution contains uncleaved $3CL^{pro}$_GST, $PreScission^{pro}$ and GST. **(C)** SDS 15% Gel of $3CL^{pro}$ after size exclusion chromatography. **(D)** $3CL^{pro}$ Chromatogram after size exclusion chromatography. **(E)** CD spectrum of $3CL^{pro}$.

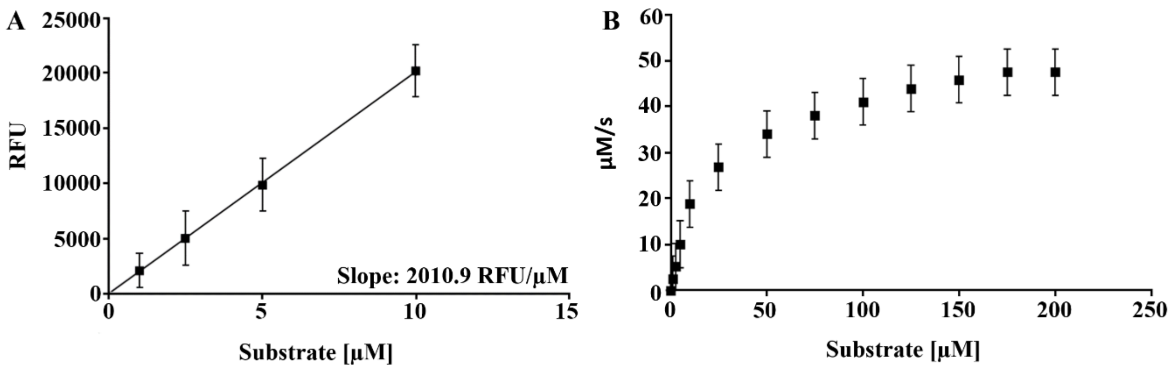

Figure S2. K_M determination of SARS-CoV-2 $3CL^{pro}$. (A) Standard curve to convert RFU to the amount of the cleaved substrate (µM). **(B)** Michaelis–Menten plot of 0.5 µM protein with various concentrations of FRET substrate. Curve fitting with Michaelis-Menten equation gave the best fitting values of K_M and V_{max} as 25.47 ± 3.43 µM and 47.52 ± 2.91 µM/s, respectively. Data shown are the mean ± SD from three independent measurements (n=3).

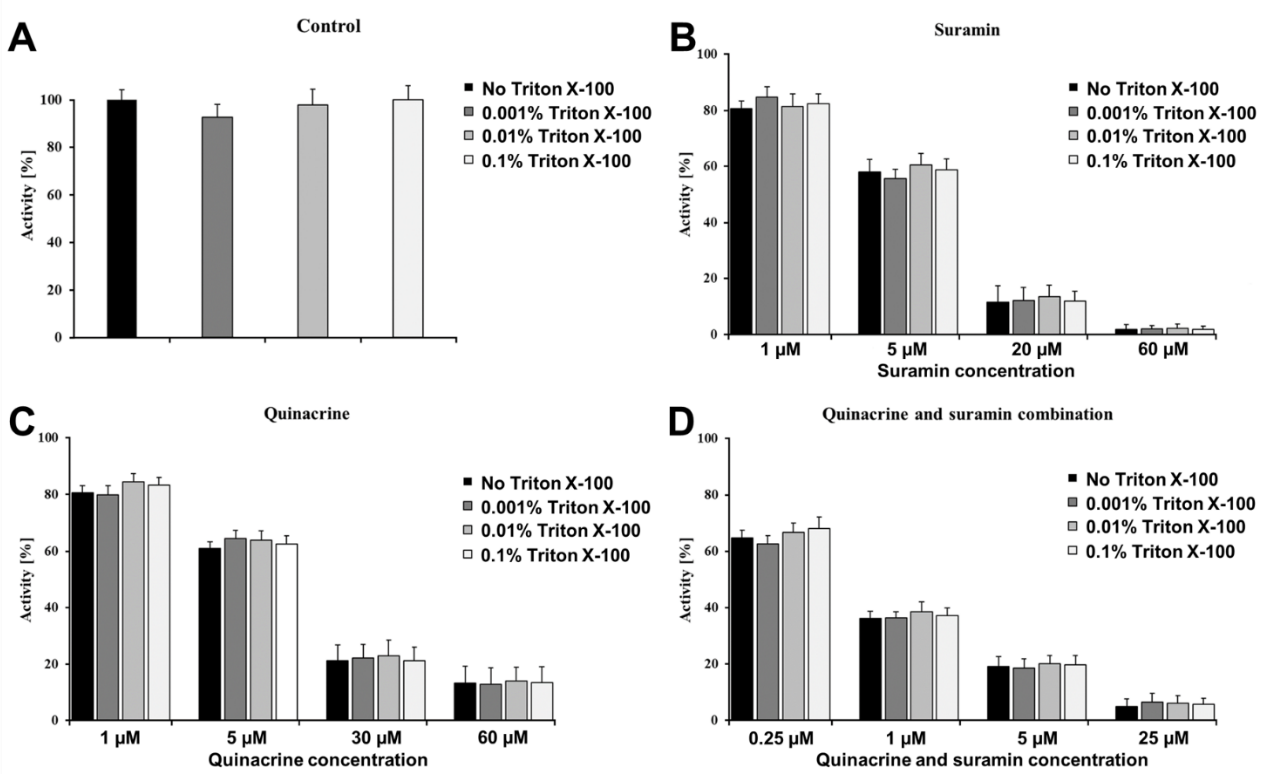

Figure S3. Effect of Triton X-100 on the Quinacrine and Suramin inhibition against SARS-CoV-2 3CL^{pro}. The experiments were performed to exclude the possibility that the molecules inhibit the protease promiscuously by aggregation. Three Triton X-100 concentrations (0.001%, 0.01% and 0.1%) were tested with four different inhibitor concentrations and compared with the results without detergent. Additionally, the effect of Triton X-100 against the protease was tested. Data shown are the mean ± SD from three independent measurements (n=3). **(A)** Effect of Triton X-100 on 3CL^{pro} activity. **(B)** Effect of Triton X-100 on Suramin inhibition against 3CL^{pro}. **(C)** Effect of Triton X-100 on Quinacrine inhibition against 3CL^{pro}. **(D)** Effect of Triton X-100 on Quinacrine and Suramin (combination) inhibition against 3CL^{pro}.

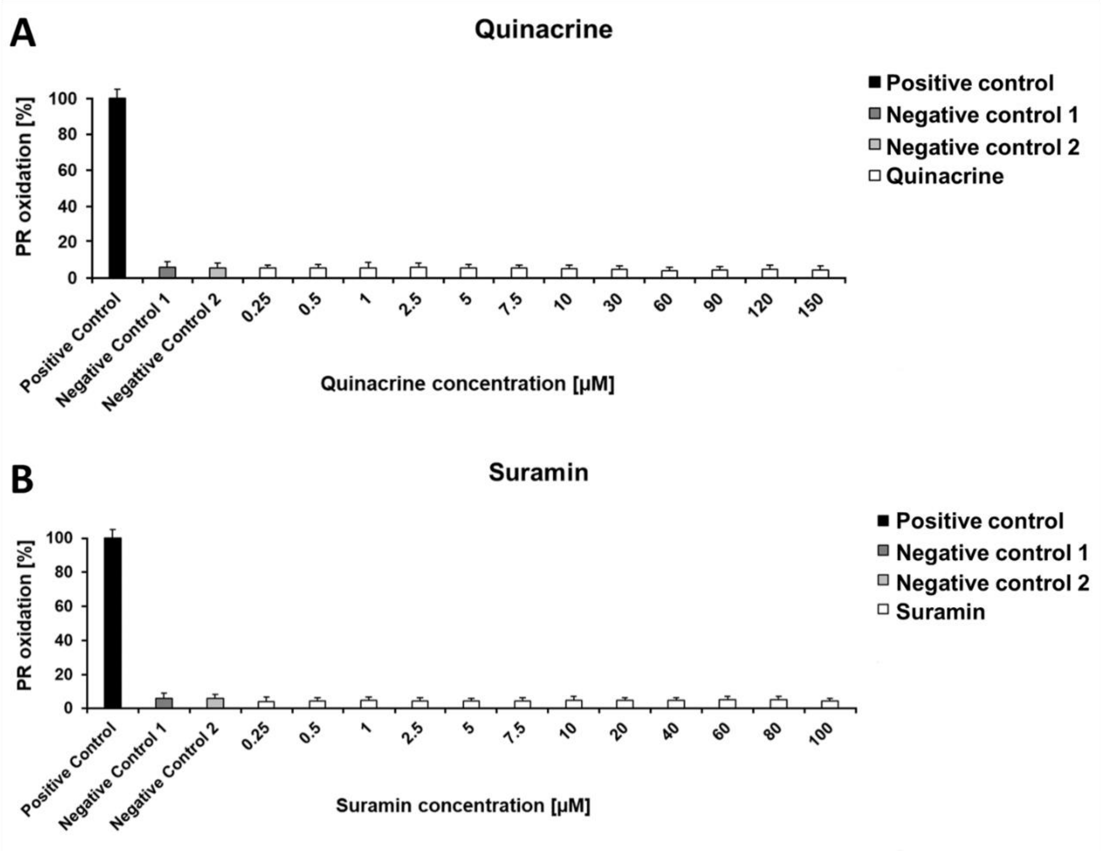

Figure S4. H_2O_2 generating capacity of Quinacrine and Suramin under the influence of 1mM TCEP. The experiments were performed to measure the H_2O_2 generating capacity of Quinacrine and Suramin under the influence of 1mM TCEP. Further, the H_2O_2-dependent horseradish peroxidase (HRP) mediates the oxidation of phenol red (PR), which can be followed at 610 nm. Positive control: HRP-PR and H_2O_2; negative control 1: HRP-PR; negative control 2: PR; Data showed are the mean ± SD from three independent measurements (n=3). **(A)** H_2O_2 generation by Quinacrine. **(B)** H_2O_2 generation by Suramin.

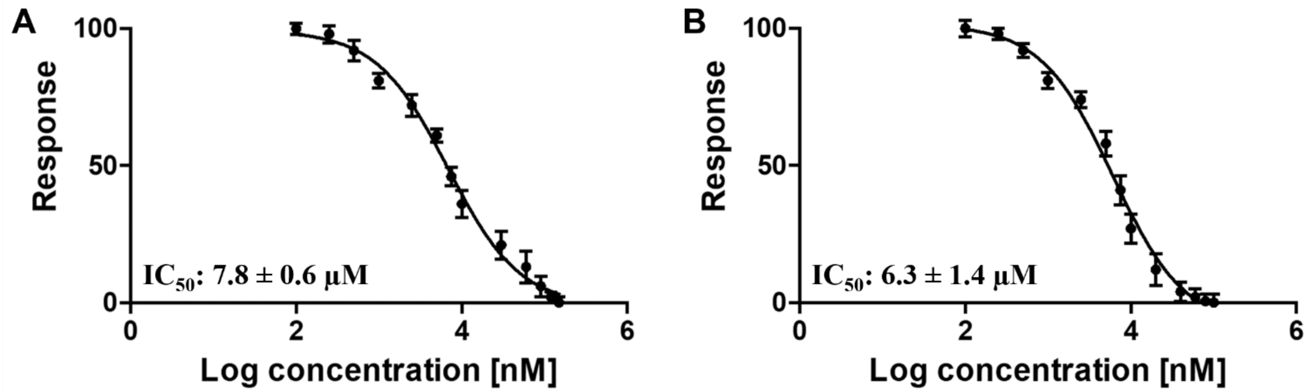

Figure S5. Quinacrine and Suramin with inhibitory activity against SARS-CoV-2 3CL^{pro}. Dose response curves for IC_{50} determination. The normalized response [%] of SARS-CoV-2 3CL^{pro} is plotted against the Log of the Quinacrine and Suramin concentration. The determined IC_{50} values are presented within the corresponding picture. **(A)** Dose response curve of Quinacrine and SARS-CoV-2 3CL^{pro}. **(B)** Dose response curve of Suramin and SARS-CoV-2 3CL^{pro}. Data shown are the mean ± SD from three independent measurements (n=3).

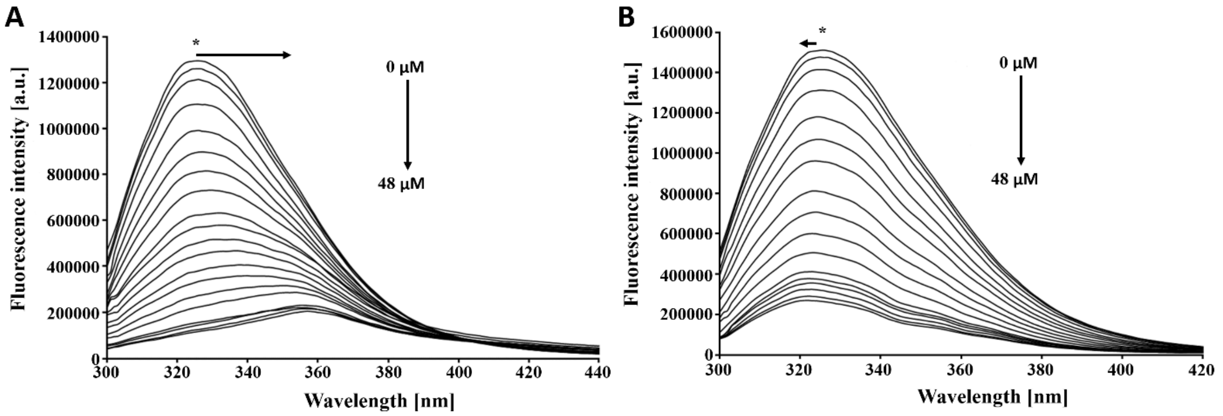

Figure S6. Fluorescence spectroscopy of Trp at 295 nm of SARS-CoV-2 3CL^{pro} in the presence of the Suramin and Quinacrine. (A) Fluorescence of SARS-CoV-2 3CL^{pro} under influence of Suramin titration demonstrated a red excitation shift of visible Trp **(B)** Fluorescence of SARS-CoV-2 3CL^{pro} under influence of Quinacrine titration demonstrated a blue excitation shift of visible Trp (*).

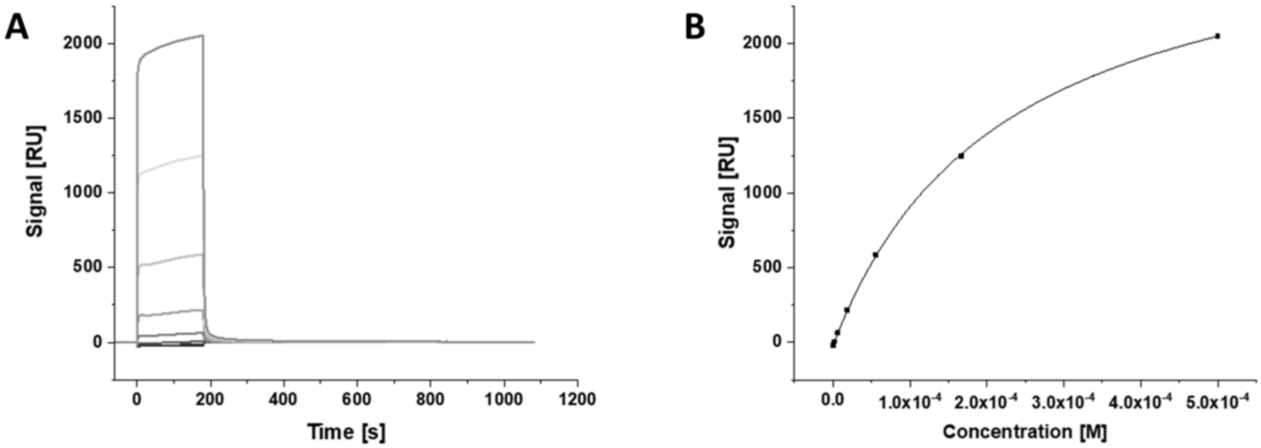

Figure S7. Equilibrium dissociation constant (K_D) determination of Quinacrine binding to SARS-CoV-2 3CLpro using surface plasmon resonance (SPR). (A) SPR sensorgram of $3CL^{pro}$ and Quinacrine. **(B)** Saturation curve for the Quinacrine and $3Cl^{pro}$ interaction.

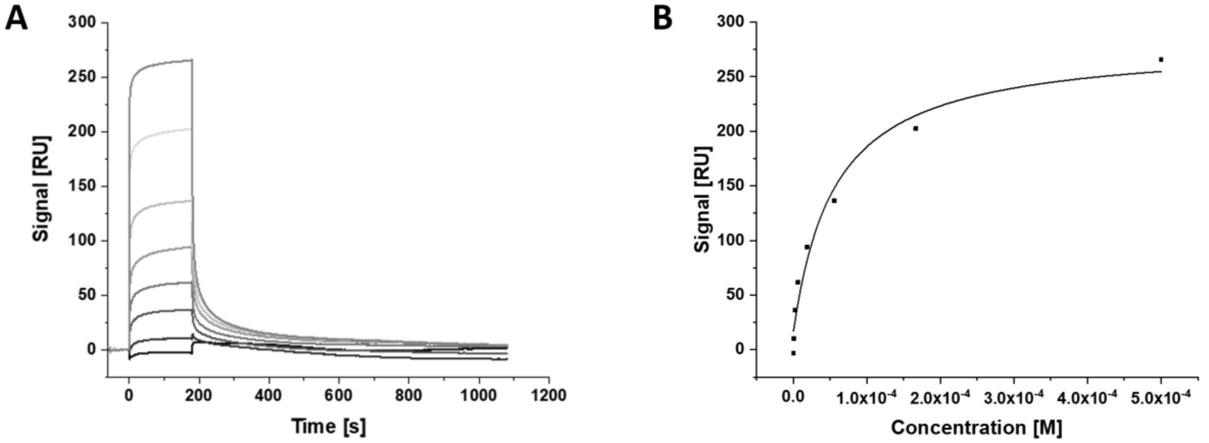

Figure S8. Equilibrium dissociation constant (K_D) determination of Suramin binding to SARS-CoV-2 $3CL^{pro}$ using surface plasmon resonance (SPR). (A) SPR sensorgram of $3CL^{pro}$ and Suramin. **(B)** Saturation curve for the Quinacrine and $3Cl^{pro}$ interaction.

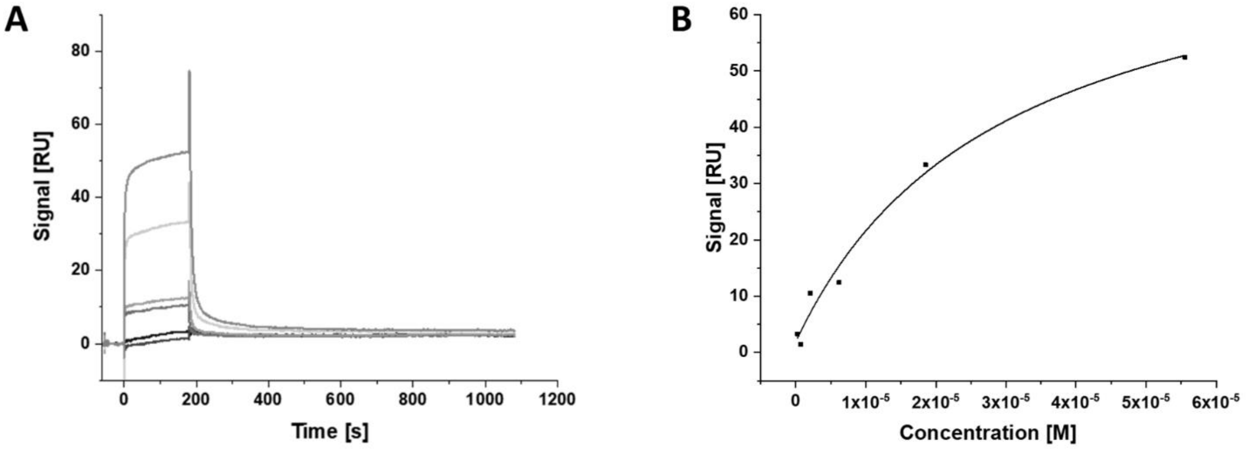

Figure S9. Equilibrium dissociation constant (K_D) determination of Quinacrine binding to SARS-CoV-2 $3CL^{pro}$ under Suramin influence using surface plasmon resonance (SPR). (A) SPR sensorgram of $3CL^{pro}$ and Quinacrine under influence of Suramin. **(B)** Saturation curve for the Quinacrine and $3Cl^{pro}$ interaction.

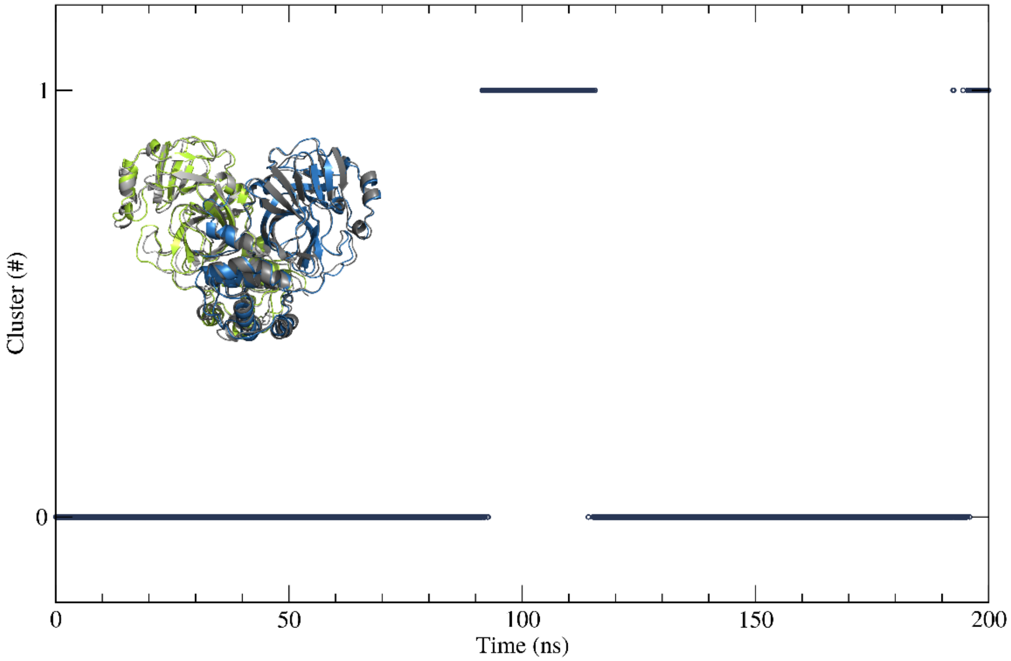

Figure S10. Clustering analysis of the SARS-CoV-2 3CL^{pro} structure after MD simulation. Pure protein pointed to a chosen structure (cluster 0) that was representative for over 86,1% of the simulation and appeared around 169 ns. The representative structure (C0) is in green and blue, while the (C1) is colored in grey.

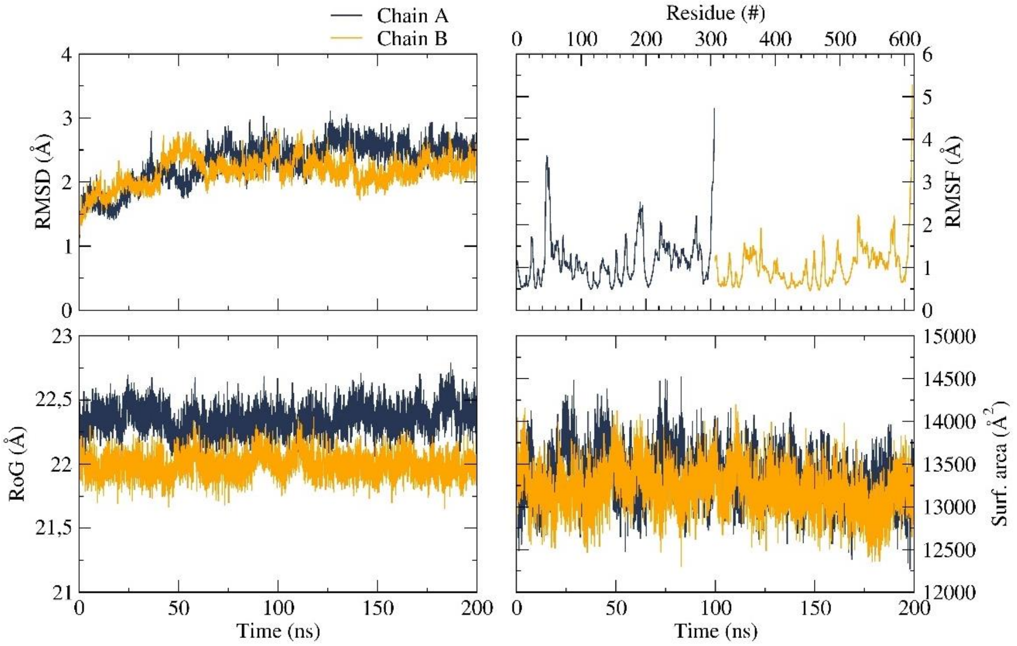

Figure S11. Time dependent modifications of the SARS-CoV-2 3CL^{pro} dimer. Protomer 1 (blue) and protomer 2 (yellow). RMSD, RoG and surface area as function of time. RMSF for each amino acid.

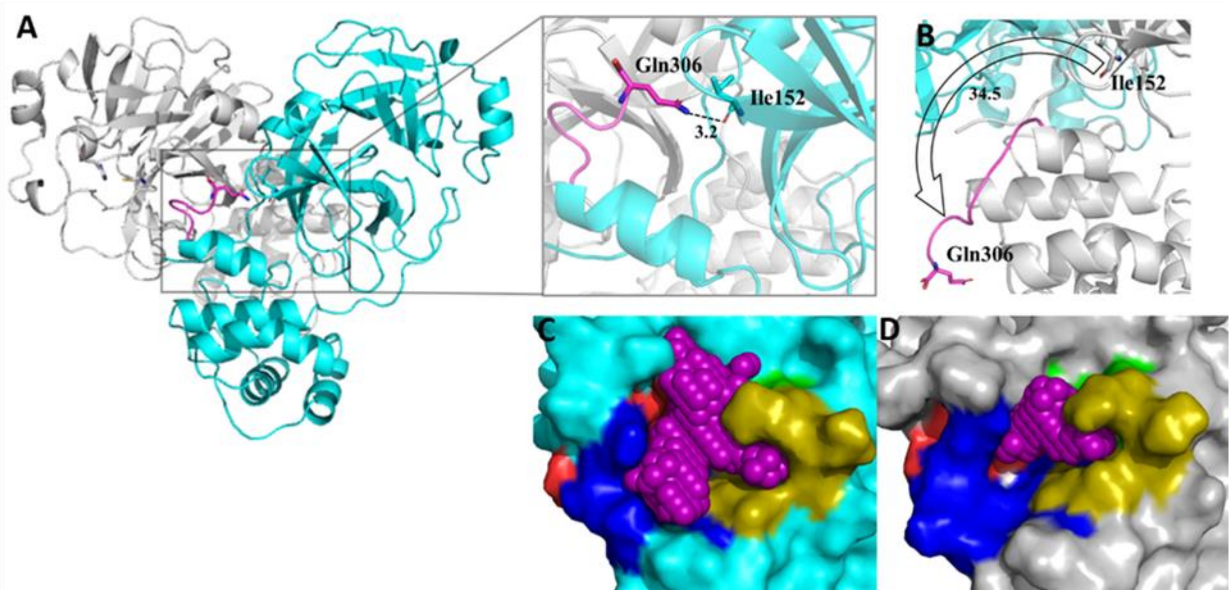

Figure S12. SARS-CoV-2 3CL^{pro} dimeric structure after MD simulation. (A) SARS-CoV-2 3CL^{pro} in ribbon view, protomer 1 in turquois and protomer 2 in grey. H-bond between C-terminus Gln306 and Ile152 in protomer 1 is highlighted. **(B)** Protomer 2 Gln306 and Ile152 develop no H-bond. **(C)** Protomer 1 active site pocket volume yields around 261 Å^3, the substrate binding subsites are highlighted. Green S1`; gold S1; red S2 and blue S3. **(D)** Protomer 2 active site volume yields about 142 Å^3.

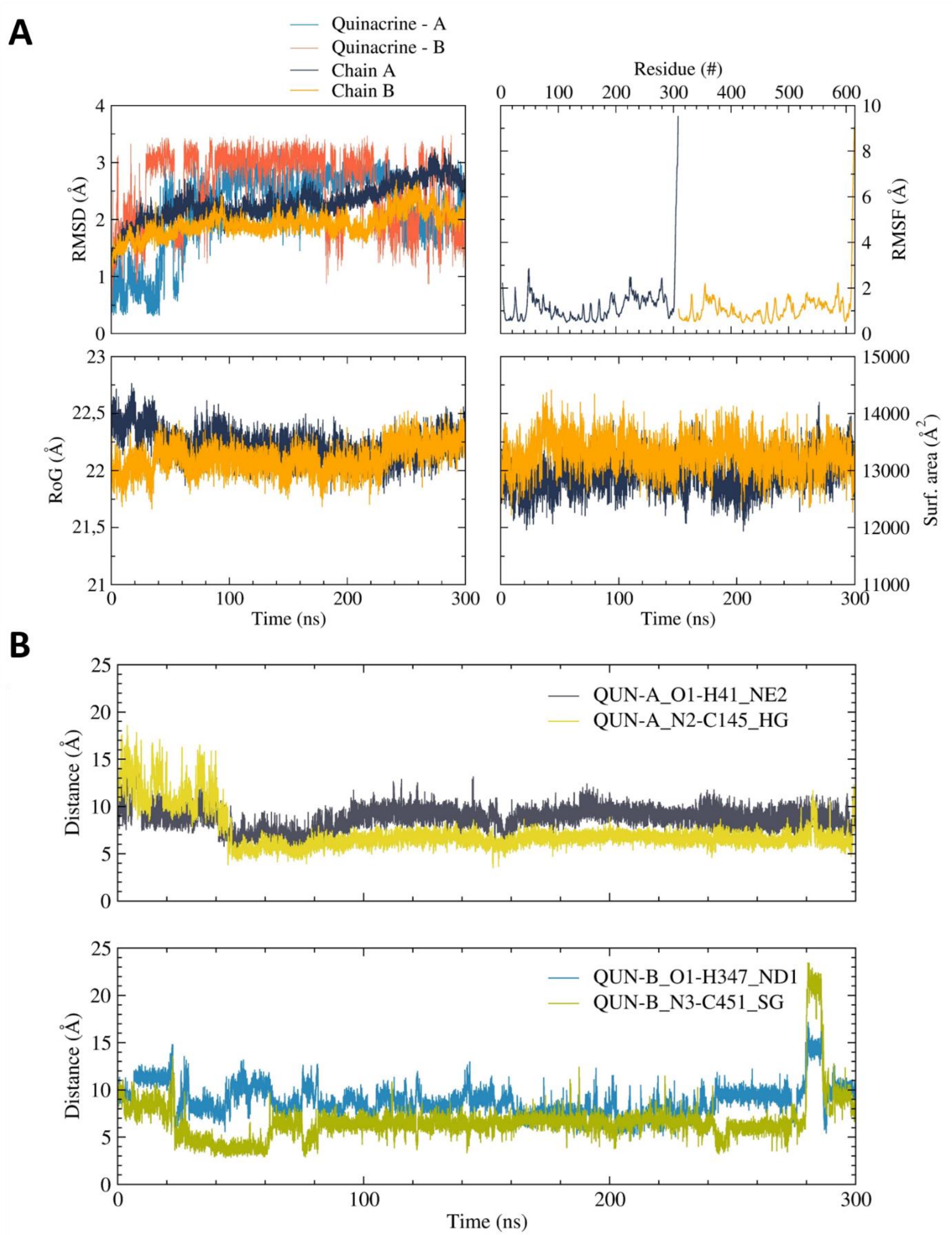

Figure S13. Time dependent modifications of SARS-CoV-2 3CL^{pro} dimer in the presence of Quinacrine in each active site. (A) Protomer 1 (blue), Quinacrine A (light blue), protomer 2 (yellow) and Quinacrine B (pink). RMSD, RoG and surface area as function of time. RMSF for each amino acid. **(B)** Distance over time of Quinacrine A and B in the protease active site.

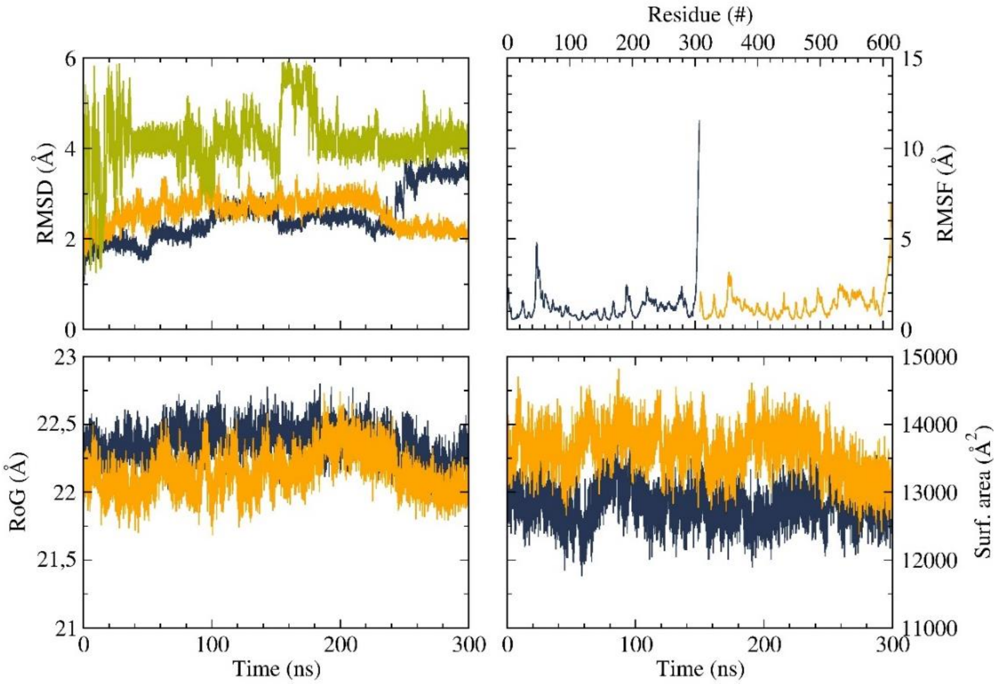

Figure S14. Time dependent modifications of SARS-CoV-2 3CL^{pro} dimer in the presence of Suramin. Protomer 1 (blue), protomer 2 (yellow) and Suramin (green). RMSD, RoG and surface area as function of time. RMSF for each amino acid.

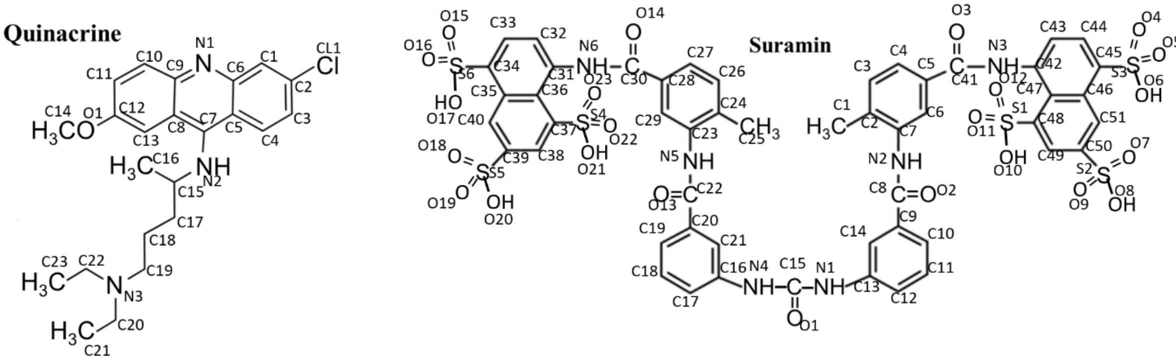

Figure S15. Atom numbers of Quinacrine and Suramin.

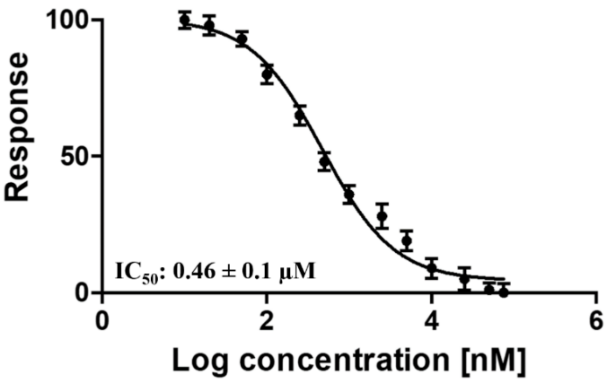

Figure S16. Quinacrine and Suramin combination with inhibitory activity against SARS-CoV-2 3CLpro. Dose response curve for IC_{50} determination. The normalized response [%] of SARS-CoV-2 3CLpro is plotted against the Log of the combined Quinacrine and Suramin concentration. The determined IC_{50} value is presented within the figure. Data shown are the mean ± SD from three independent measurements (n=3).

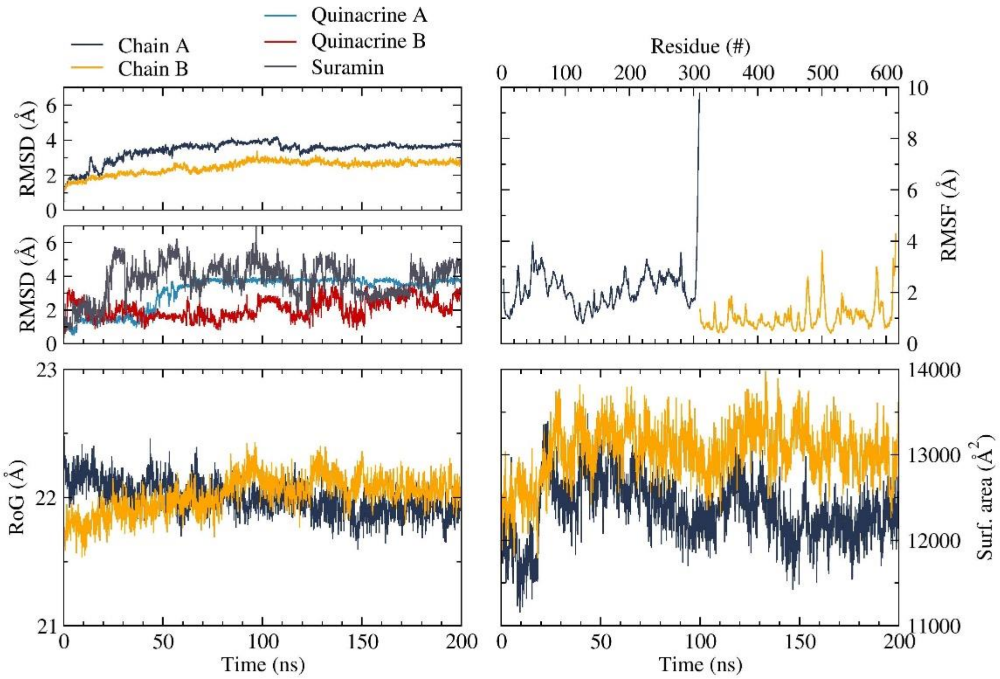

Figure S17. Time dependent modifications of SARS-CoV-2 3CLpro dimer in the presence of Quinacrine and Suramin. Protomer 1 (blue), protomer 2 (yellow), Suramin (gray), Quinacrine A (light-blue), Quinacrine B (red). RMSD, RoG and surface area as function of time. RMSF for each amino acid.

Table S1. K_D values of Quinacrine and Suramin SARS-CoV-2 $3CL^{pro}$, determined by SPR.

Molecule	Experiment 1 K_D [µM]	Experiment 2 K_D [µM]	Experiment 3 K_D [µM]	Average K_D [µM]	STD [µM]
**Quinacrine**	233.5	222.3	-	227.9	± 7.9
**Suramin**	62.9	56.5	-	59.7	± 4.5
**Quinacrine***	15.05	45.9	29.6	30.2	± 12.7

*K_D determination of Quinacrine to SARS-CoV-2 $3CL^{pro}$ in the presence of Suramin.

Table S2. Contributing amino acids in the Substrate-binding site of SARS-CoV-2 $3CL^{pro}$.

Substrate subsite*	SARS-CoV-2 $3CL^{pro}$
**S1`**	His41
	Gly143
	Ser144
	Cys145
**S1**	Phe140
	Leu141
	Asn142
	His163
	Glu166
**S2**	His41
	Met49
	Tyr54
	His164
	Asp187
	Arg188
**S3**	Met165
	Glu166
	Leu167
	Gln189
	Thr190
	Gln192
**Oxyanion hole**	His41
	Cys145

* Based on Tang et al. 2020 [1]

Table S3. SARS-CoV-2 $3CL^{pro}$ Residues involved in forming H-bonds and hydrophobic contacts with Quinacrine, Suramin and Quinacrine + Suramin.

Ligand	Interacting residues	
	H-Bond	*Hydrophobic interaction*
**Quinacrine**	Met165, Gln189	His41, Met49, Val186, Asp187, Arg188, Gln194
**Suramin**	Lys12, Lys97, Lys100, Tyr101, Phe103	Lys97, Lys100, Lys102, Phe103, Val104, Arg105
**Quinacrine (+Suramin)**	Thr45	Thr25, His41, Cys44, Thr45, Met49, Pro52
**Suramin (+Quinacrine)**	Lys12, Thr98, Lys97`	Pro96, Lys97, Thr98, Tyr 101

Table S4. Affected proteins by mutations in SARS-CoV-2 variants.

Variant	Origin	Orf1ab	Orf3	Orf8	S[1]	E[2]	**M[3]**	N[4]	D[5]
**B.1.1.7**	UK	T1001I<br>A1708D<br>I2230T		Q27<br>Y73C<br>R52I	N501Y<br>A570D<br>P681H<br>T716I<br>S982A<br>D1118H			D3L<br>S235F	11288:9<br>21765:6<br>21991:3
**B.1.351**	South Africa	K1655N			D80A<br>D215G<br>K417N<br>E484K<br>N501Y<br>A701V	P71L		T205I	
**P.1**	Brazil	S1188L<br>K1795Q	G174C	E92K	L18F<br>T20N<br>P26S<br>D138Y<br>R190S<br>K417T<br>E484K<br>N501Y<br>H655Y<br>T1027I			P80R	11288:9
**A.23.1**	Uganda				F157L<br>V367F<br>Q613H<br>P681R				
**B.1.525**	UK, Nigeria	L4715F			E484K<br>Q52R<br>Q677H<br>F888L	L21F<br>I82T			11288:9<br>21765:6<br>28278:3
**B.1.526[2]**	USA	T226I<br>P861L<br>Q876H<br>L3202P<br>T3953I<br>P4716L<br>Q5413H			L5F<br>T95I<br>D253G<br>E484K<br>D614G<br>A701V			P199L<br>M234I	
**B.1.617[3]**	India	T1567I<br>T3646A<br>P4716L<br>G5530C<br>M5753I<br>K6711R<br>S6713A			T95I<br>G142D<br>E154K<br>L452R<br>E484Q<br>D614G<br>P681R<br>Q1071H		I82S	R203M<br>D377Y	

[1] Spike glykoprotein

[2] Envelope protein

[3] Membrane

[4] N-terminus

[5] Deletion

[2] Based on West et al. 2021

[3] Based on Yadav et al. 2021

References

[1] Tang, B.; He, F.; Liu, D.; Fang, M.; Wu, Z.; Xu, D. AI-aided design of novel targeted covalent inhibitors against SARS-CoV-2. bioRxiv. **2020**.

[2] West Jr, A.P.; Barnes, C.O.; Yang, Z.; Bjorkman, P.J. SARS-CoV-2 lineage B. 1.526 emerging in the New York region detected by software utility created to query the spike mutational landscape. bioRxiv. **2021**.

[3] Yadav, P.D.; Sapkal, G.N.; Abraham, P.; Ella, R.; Deshpande, G.; Patil, D.Y.; Nyayanit, D.A.; Gupta, N.; Sahay, R.R.; Shete, A.M.; Panda, S.; Bhargava, B.; Mohan, V.K. Neutralization of variant under investigation B. 1.617 with sera of BBV152 vaccinees. bioRxiv. **2021**.
